# Diagnosis of major depressive disorder based on changes in multiple plasma neurotransmitters: a targeted metabolomics study

**DOI:** 10.1038/s41398-018-0183-x

**Published:** 2018-07-10

**Authors:** Jun-Xi Pan, Jin-Jun Xia, Feng-Li Deng, Wei-Wei Liang, Jing Wu, Bang-Min Yin, Mei-Xue Dong, Jian-Jun Chen, Fei Ye, Hai-Yang Wang, Peng Zheng, Peng Xie

**Affiliations:** 1 Department of Neurology, Yongchuan Hospital, Chongqing Medical University, Chongqing, 402460 China; 2 Chongqing Key Laboratory of Neurobiology, Chongqing, 400016 China; 3 Institute of Neuroscience and the Collaborative Innovation Center for Brain Science, Chongqing Medical University, Chongqing, 400016 China; 4 The M.O.E. Key Laboratory of Laboratory Medical Diagnostics, the College of Laboratory Medicine, Chongqing Medical University, Chongqing, 400016 China; 5 Department of Neurology, The First Affiliated Hospital of Chongqing Medical University, Chongqing, China

## Abstract

Major depressive disorder (MDD) is a debilitating psychiatric illness. However, there is currently no objective laboratory-based diagnostic tests for this disorder. Although, perturbations in multiple neurotransmitter systems have been implicated in MDD, the biochemical changes underlying the disorder remain unclear, and a comprehensive global evaluation of neurotransmitters in MDD has not yet been performed. Here, using a GC-MS coupled with LC-MS/MS-based targeted metabolomics approach, we simultaneously quantified the levels of 19 plasma metabolites involved in GABAergic, catecholaminergic, and serotonergic neurotransmitter systems in 50 first-episode, antidepressant drug-naïve MDD subjects and 50 healthy controls to identify potential metabolite biomarkers for MDD (training set). Moreover, an independent sample cohort comprising 49 MDD patients, 30 bipolar disorder (BD) patients and 40 healthy controls (testing set) was further used to validate diagnostic generalizability and specificity of these candidate biomarkers. Among the 19 plasma neurotransmitter metabolites examined, nine were significantly changed in MDD subjects. These metabolites were mainly involved in GABAergic, catecholaminergic and serotonergic systems. The GABAergic and catecholaminergic had better diagnostic value than serotonergic pathway. A panel of four candidate plasma metabolite biomarkers (GABA, dopamine, tyramine, kynurenine) could distinguish MDD subjects from health controls with an AUC of 0.968 and 0.953 in the training and testing set, respectively. Furthermore, this panel distinguished MDD subjects from BD subjects with high accuracy. This study is the first to globally evaluate multiple neurotransmitters in MDD plasma. The altered plasma neurotransmitter metabolite profile has potential differential diagnostic value for MDD.

## Introduction

Major depressive disorder (MDD) is a common mental illness, with more than 300 million people of all ages affected worldwide, according to a report by the World Health Organization in 2017^1^. MDD dramatically reduces the quality of life of the affected individuals, and causes them to function poorly at work, at school and in the family, and can also lead to suicide. MDD result from a complex interaction of social, psychological, and biological factors^2,3^. Despite extensive researches, the molecular biology mechanisms of depression remain poorly understood. Currently, diagnosis of MDD primarily relies on subjective identification of symptom clusters by psychiatrists, resulting in a high rate of misdiagnosis^4–6^. Because of the lack of an objective diagnostic method, fewer than half of MDD patients (in many countries, fewer than 10%) receive effective treatments^7,8^. Thus, an objective diagnostic approach for MDD would be of considerable clinical value.

Our group has focused on MDD over a decade, and has previously conducted proteomics and non-targeted metabolomics studies on rodent models of depression, on non-human primate (Macaca fascfeiicularis) model, and on patients with MDD^9–15^. Previous studies have showed that perturbations in central and peripheral neurotransmitters are a hallmark of MDD. In particular, MDD patients showed disturbances in several neurotransmitters in the periphery and brain, including dopamine, glutamate, γ-aminobutyric acid (GABA), and serotonin (5-HT)^5,16–18^ which were thought to be involved in the pathogenesis of the disorder. These studies suggest that numerous neurotransmitters are perturbed in individuals with MDD. However, a comprehensive evaluation of neurotransmitter levels in depression has not yet been performed.

Brain tissues and cerebrospinal fluid are ideal biological samples for research on neuropsychiatric disorders^19,20^. However, brain tissue biopsy and lumbar puncture samples cannot be practically obtained from depressed patients because of ethical and safety concerns. In comparison, blood samples can be acquired at minimal risk and cost, and are commonly used in clinical laboratories^21,22^. Thus, a plasma-based diagnostic test for MDD would be clinically practical. In addition, peripheral metabolic disturbances have been found in MDD, suggesting that characteristic metabolic alterations associated with the pathogenesis of MDD may generate a detectable molecular phenotype in the blood for diagnosis^23,24^. Thus, plasma samples were used in the present study.

Here, the applicability of a plasma-targeted metabonomic method for the diagnosis of MDD was evaluated. A total of 19 neurotransmitters and relevant metabolites were quantified by gas chromatography-mass spectrometry (GC-MS) coupled with liquid chromatography-tandem mass spectrometry (LC-MS/MS). This approach was used to distinguish 50 first-episode, antidepressant drug-naïve depressed patients from 50 healthy controls. This method can reliably detect metabolites involved in the GABAergic, catecholaminergic and serotonergic systems in plasma. We sought to characterize metabolite changes in the early stage of MDD with the aim of identifying potential diagnostic biomarkers for the disorder. In addition, an independent sample cohort, comprising 49 unselected MDD patients, 30 bipolar disorder (BD) patients, and 40 healthy controls, was used to validate the diagnostic performance of the biomarkers.

## Materials and methods

### Ethics statement

The protocols of this study were reviewed and approved by the Ethical Committee of Chongqing Medical University. Prior to sample collection, written informed consent was acquired from all recruited subjects. All procedures were conducted according to the principles expressed in the Declaration of Helsinki.

### Participants

Totally, 99 MDD patients and 30 BD patients were recruited from the psychiatric center of the First Affiliated Hospital of Chongqing Medical University. All diagnoses were performed by two experienced psychiatrists according to the Structured Psychiatric Interview using DSM-IV-TR criteria as in our previous studies^5,25^. The MDD and BD subjects with pre-existing physical or other mental disorders, or illicit drug abuse, pregnancy, nursing, or menstruation for female subjects were excluded. The 17-item Hamilton Depression Rating Scale (HDRS) was applied to assess the severity of MDD. During the same time period, 90 healthy controls were recruited from the same site and were required to have no current or previous lifetime history of neurological, DSM-IV Axis I/II diagnosis, systemic medical illness and family history of any psychiatric disorders.

The recruited depressed patients and healthy controls were divided into two cohorts. In Cohort 1 (training set), relatively high homogeneous samples including 50 first-episode, antidepressant drug-naïve MDD subjects and 50 demographically matched healthy controls were used to identify candidate metabolite biomarkers for MDD. In Cohort 2 (testing set), 49 unselected MDD patients (11 unmedicated MDD samples and 38 medicated MDD samples) and 40 healthy controls were used to independently validate the diagnostic generalizability of the biomarkers. Moreover, 30 BD patients (11 unmedicated BD samples and 19 medicated BD samples) were recruited in Cohort 2 to assess the diagnostic specificity of the plasma metabolite biomarkers. There were two reasons accounting for this choice: (i) some clinical symptoms of BD overlapped with MDD and (ii) our previous studies had shown that BD were associated with disturbances of peripheral neurotransmitter metabolites.

### Targeted metabolomic analysis

Sample preparation, and GC-MS and LC-MS/MS analysis were performed as our previous studies^25,26^. Briefly, plasma samples were extracted and analyzed on GC-MS and LC-MS/MS. For GC/MS, samples were analyzed on an Agilent 7890 A/5975 C Inert Triple Axis Detector (Agilent, USA). The original spectral data from GC−MS were converted to NetCDF format and then processed by XCMS software for peak finding, integration and alignment. LC-MS/MS analysis was employed to quantify low abundance neurotransmitters, using a Waters ACQUITY UPLC and AB Sciex Triple Quad6500 mass spectrometry system. Data collection and analysis for LC-MS/MS were performed using Analyst software (AB Sciex, v. 1.5.2) on the default parameters for automatic identification and integration of the MRM transition. The additional details information of targeted metabolomic analysis was shown in Supplemental Materials.

### Identification of plasma metabolite biomarkers for MDD

As clinical diagnosis based on the quantification of a small number of metabolites would be more practical, a binary logistic regression analysis was used to optimize the metabolite biomarker combination. To evaluate the diagnostic generalizability of the MDD biomarkers, the ability of the simplified biomarker panel to discriminate MDD subjects from non-MDD subjects was quantified using receiver-operating characteristic (ROC) curve analysis.

### Statistical analysis

The chi-square test was used to analyze categorical data (sex). All continuous variables such as age, BMI and metabolite concentrations, were analyzed using Student’s two-tailed *t*-test or one-way ANOVA followed by the Bonferroni post hoc test. All continuous variables were expressed as means ± standard errors of the mean. All analyses were performed with MedCalc v. 15.2.1 (MedCalc Software, Mariakerke, Belgium). A *p*-value of less than 0.05 was considered statistically significant. Heat maps of the metabolites were obtained using MetaboAnalyst 3.0 (http://www.metaboanalyst.ca/)^27^. This web server is designed to permit comprehensive metabolomic data analysis, visualization, and interpretation.

## Results

### Clinical information of the recruited subjects

50 first-episode, antidepressant drug-naïve MDD subjects and 50 demographically matched healthy controls were divided into cohort 1 and used to identify candidate metabolite biomarkers for MDD. Medicated and unmedicated MDD and BD subjects were recruited in cohort 2 and were used to independently validate diagnostic generalizability of identified biomarkers, which paves the way for translating the identified biomarkers for clinical practice. All depressed patients scored higher on the HDRS than healthy controls in both cohort 1 and cohort 2. Demographic parameters such as age, gender and BMI did not differ among the groups in either cohort 1 or cohort 2. The key clinical characteristics of the recruited subjects were presented in Table 1.

**Table 1:** Demographic characteristics of the recruited subjects

Variables	Cohort 1			Cohort 2			
	HC	MDD	P^a^	HC	MDD	BD	P^a^
Sample size	50	50	–	40	49	30	–
Sex (M/F)	25/25	24/26	0.841	22/18	23/26	13/17	0.593
Age (years) ^b^	36.9±1.3	38.3±1.6	0.503	36.8±1.6	37.7±1.7	35.8±10.7	0.249
BMI^b^	22.4±0.73	22.0±0.39	0.553	21.7±0.7	22.5±0.7	22.4±3.4	0.264
HDRS scores	0.4±0.1	24.6±0.5	<0.01	0.3±0.1	23.3±0.5	16.7±10.5	<0.01
BD-I	–	–	–	–	–	18	–
BD-II	–	–	–	–	–	12	–
Course (Month)	–	17.4±2.2	–	–	41.6±9.8	64.9±15.1	–
Medication (Y/N)	N	N	–	–	38/11	19/11	–
SSRI(Y/N)	N	N	–	N	29/20	10/20	–
SNRI (Y/N)	N	N	–	N	9/40	N	–
Mood stabilizers (Y/N)	N	N	–	N	N	5/25	–
Atypical antipsychotics(Y/N)	N	N	–	N	N	4/26	–

*HC* healthy controls, *MDD* major depressive disorder, *BD* bipolar disorder, *Y/N* Yes/No, *M/F* male/female, *HDRS* Hamilton depression rating scale, *BMI* body mass index, *SSRI* selective serotonin reuptake inhibitors, *SNRI* serotonin noradrenalin reuptake inhibitors

^a^ Two-tailed Student’s test or one-way ANOVA for continuous variables (age, BMI, and HDRS scores); Chi-square analysis was used for categorical variables (sex)

^b^ Values were expressed as mean ± SEM

### Characterization of differentially expressed neurometabolites between MDD subjects and healthy controls

**Fig. 1:**
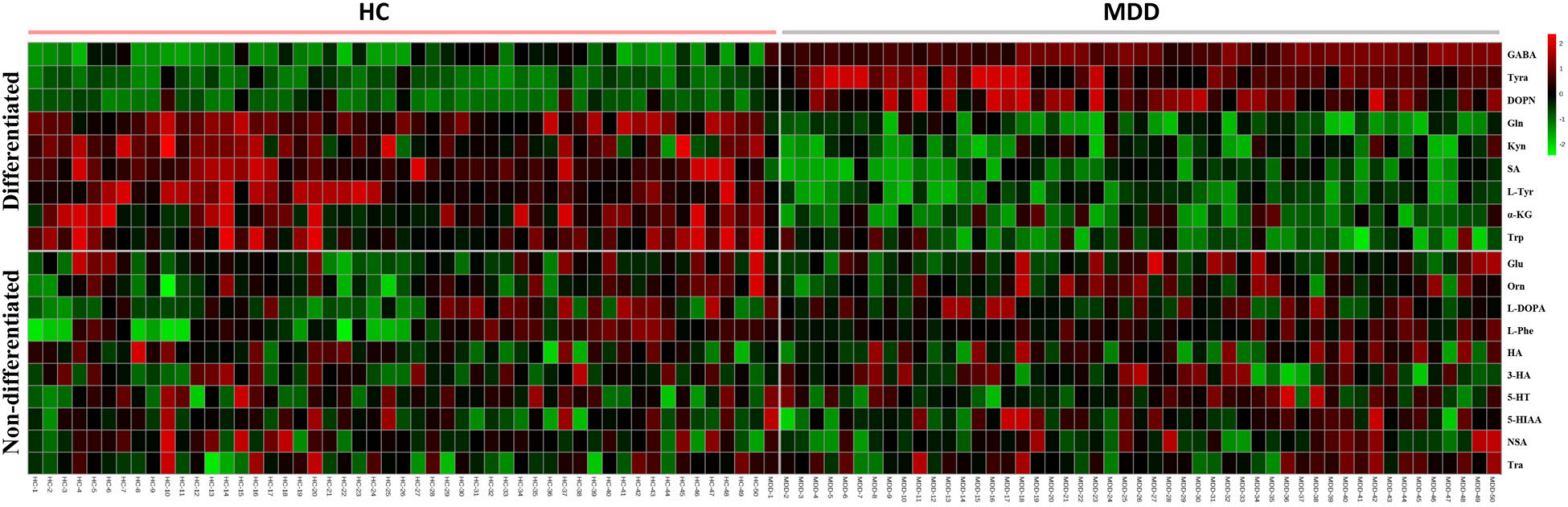
Heat map of plasma neurometabolites in MDD subjects and healthy controls. SA succinic acid, GABA γ-aminobutyric acid, a-KG α-ketoglutaric acid, Gln glutamine, Glu glutamic acid, Orn ornithine, l-Tyr l-tyrosine, Tyra tyramine, DOPN dopamine, L-DOPA L-3,4-dihydroxyphenylalanine, l-Phe l-phenylalanine, HA homovanillic acid, Trp tryptophan, Kyn kynurenine, 3-HA 3-hydroxyanthranilic acid, 5-HT 5-hydroxytryptamine, 5-HIAA 5-hydroxyindoleacetic acid, NAS N-acetyl-serotonin, Tra tryptamine. The heat map was generated using MetaboAnalyst 3.0 (www.metaboanalyst.ca) for each metabolite

Initially, to uncover how the three metabolic pathways changed in the early stage of MDD, levels of 19 nuermetabolites in first-episode, antidepressant drug-naïve MDD patients were compared with healthy controls. The neurometabolite concentration was obtained from the mass peak area of the sample analyte. Two-tailed Student’s *t*-test showed significant differences in plasma neurometabolites between the two groups (Table 2, Fig. 1). The analysis revealed that 9 of 18 plasma metabolites were significantly changed. Three metabolites—γ-aminobutyric acid (GABA), tyramine (Tyra), and dopamine (DOPN)—were significantly increased in MDD subjects relative to healthy controls. Moreover, the levels of six metabolites—succinic acid (SA), α-ketoglutaric acid (a-KG), glutamine (Gln), L-tyrosine (L-Tyr), tryptophan (Trp), and kynurenine (Kyn)—were significantly decreased in MDD subjects relative to healthy controls

**Table 2:** Concentration (ng/g) of plasma neurometabolites in cohort 1

Metabolites	Platform	Metabolic pathway	MDD	HC	Log_2_(FC)	*p*-value
SA	GC-MS	GABAergic	922.40±37.34	1083.14±48.10	−0.23	**0.01**
GABA	GC-MS	GABAergic	373.45±2.50	335.89±3.18	0.15	**0.000**
α-KG	GC-MS	GABAergic	10253.24±460.18	12863.54±897.06	−0.33	**0.012**
Gln	GC-MS	GABAergic	33032.90±2249.47	51223.80±2949.99	−0.63	**0.000**
Glu	GC-MS	GABAergic	15246.52±1107.83	14274.68±1024.47	0.10	0.521
Orn	GC-MS	GABAergic	15908.58±824.00	14520.46±801.36	0.13	0.23
l-Tyr	GC-MS	Catecholaminergic	8755.64±379.78	10224.73±527.16	−0.22	**0.026**
Tyra	LC-MS/MS	Catecholaminergic	19.26±4.30	1.98±0.33	3.28	**0.000**
DOPN	LC-MS/MS	Catecholaminergic	1.01±0.16	0.28±0.04	1.86	**0.000**
L-DOPA	GC-MS	Catecholaminergic	153.67±3.75	145.16±3.28	0.08	0.091
l-Phe	GC-MS	Catecholaminergic	10508.21±355.70	9671.28±538.41	0.12	0.198
HA	LC-MS/MS	Catecholaminergic	56.11±4.13	52.85±3.50	0.09	0.549
Trp	GC-MS	Serotonergic	591.01±45.51	902.68±89.38	−0.61	**0.002**
Kyn	LC-MS/MS	Serotonergic	1571.31±116.29	1992.25±78.81	−0.34	**0.004**
3-HA	GC-MS	Serotonergic	819.20±30.63	802.80±38.78	0.03	0.741
5-HT	LC-MS/MS	Serotonergic	140.27±28.80	186.63±79.81	−0.41	0.586
5-HIAA	LC-MS/MS	Serotonergic	21.30±2.03	18.31±1.17	0.22	0.206
NAS	LC-MS/MS	Serotonergic	0.77±0.11	0.85±0.11	−0.15	0.593
Tra	LC-MS/MS	Serotonergic	1.64±0.22	1.44±0.11	0.19	0.430

A negative log_2_ (FC) indicates significantly lower expression in MDD subjects compared with healthy controls. A positive log_2_ (FC) indicates significantly higher expression in MDD subjects compared with healthy controls

The data were analyzed using one-way ANOVA followed by Bonferroni’s post hoc test

Values in bold denote statistically significant differences (*p* < 0.05)

*SA* succinic acid, *GABA* γ-aminobutyric acid, *a-KG* α-ketoglutaric acid, *Gln* glutamine, *Glu* glutamic acid, *Orn* ornithine, *l-Tyr* l-tyrosine, *Tyra* tyramine, *DOPN* dopamine, *L-DOPA* L -3,4-dihydroxyphenylalanine, *l-Phe* l-phenylalanine, *HA* homovanillic acid, *Trp* tryptophan, *Kyn* kynurenine, *3-HA* 3-hydroxyanthranilic acid, *5-HT* 5-hydroxytryptamine, *5-HIAA* 5-hydroxyindoleacetic acid, *NAS* N-acetyl-serotonin, *Tra* tryptamine

### Assessment of diagnostic perfomance

To obtain a simple plasma metabolite biomarker panel that would be useful in diagnosing MDD in clinical practice, all key differential metabolites contributing to the discrimination between MDD subjects and healthy controls were used in univariate ROC curve analysis. The AUC of DOPN, GABA, Tyra, Gln, Trp, Kyn, SA, α-KG, L-Tyr, were 0.893, 0.887, 0.813, 0.771, 0.741, 0.685, 0.648, 0.637, 0.610, respectively. Among the nine neurometabolites, GABA showed the highest sensitivity (100% sensitivity), whereas the specificity was low. The sensitivity and specificity of the DOPN were 92 and 78%, respectively. The detail results of ROC curve analysis for each differential metabolite are shown in Supplemental Table 3 and Supplemental Fig. 1.

**Fig. 2:**
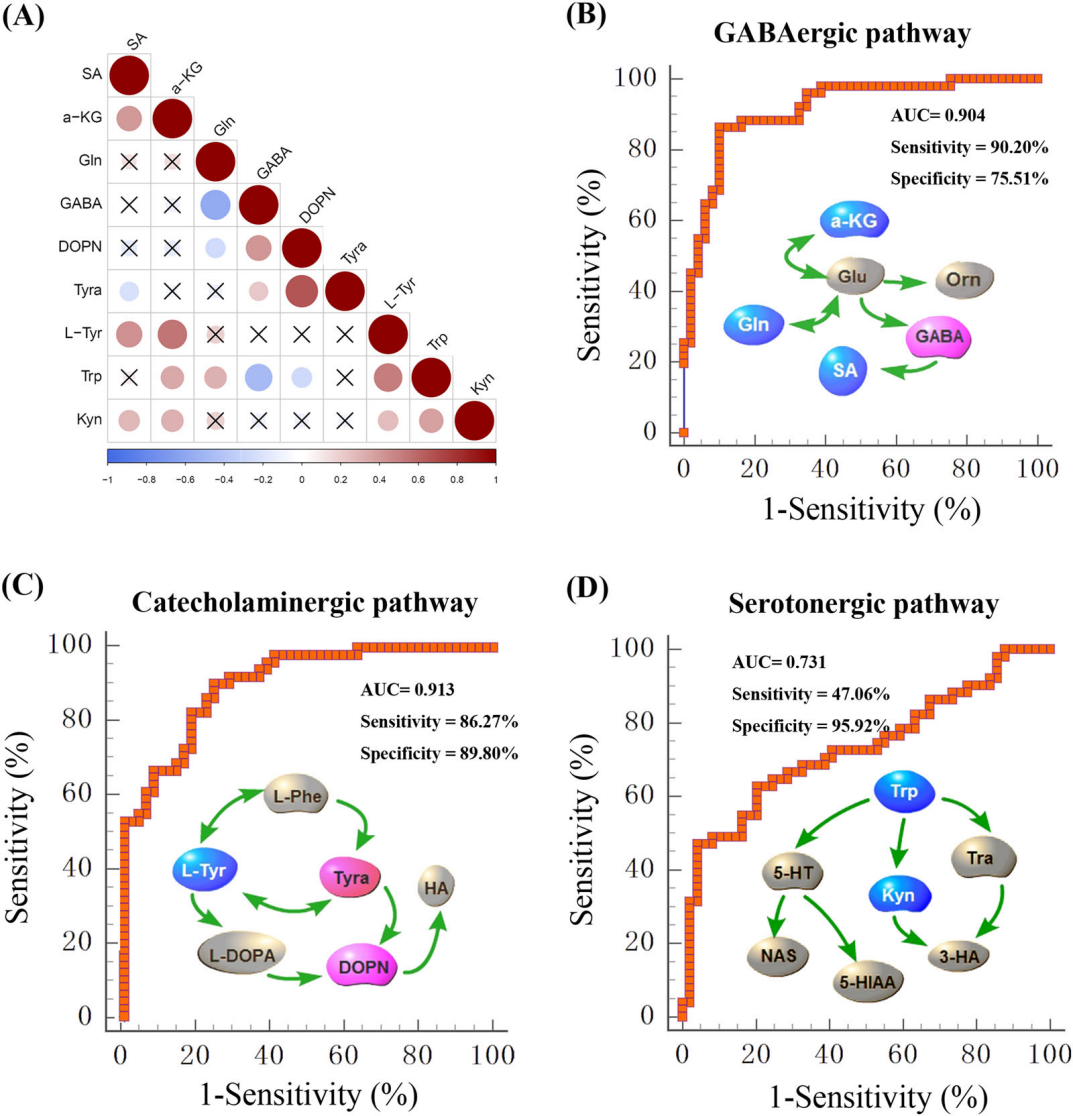
Systems analysis of differential metabolites in MDD subjects and healthy controls. **a** The correlation heatmap displays the correlation coefficients (Spearman) among differential metabolites. The color-coded scale of correlation is at the bottom, where a blue color indicates a positive correlation, while a red color indicates a negative correlation. **b**–**d** ROC curve of GABAergic, catecholaminergic, and serotonergic pathway. neurometabolite symbols with red were upregulated while blue were downregulated in MDD subjects compared with healthy controls

To investigate the relationship among the nine differential metabolites, their levels in the plasma samples from patients and healthy controls were evaluated using Spearman’s correlation (Fig. 2a, Supplemental Fig. 2). A positive correlation is indicated with a blue color, whereas a negative correlation is indicated with a red color. Remarkably, the metabolites in the same pathway showed good correlations. Next, the nine differential metabolites were divided into three groups according to their function, and used to perform ROC analysis to identify the optimal plasma metabolite biomarker panel (Fig. 2b–d). From the ROC curves, the calculated sensitivities and specificities of the three pathways in diagnosing MDD are shown in Fig. 2 and Supplemental Table 4. The sensitivities of the GABAergic, catecholaminergic, and serotonergic pathways were 90.20%, 86.27% and 47.06%, respectively, and the specificities were 75.51%, 89.80%, and 95.92%, respectively. The three ROC curves were further analyzed with McNemar’s test^28^. The GABAergic and catecholaminergic pathways were better able to diagnose MDD than the serotonergic pathway (*p* *<* 0.05).

**Fig. 3:**
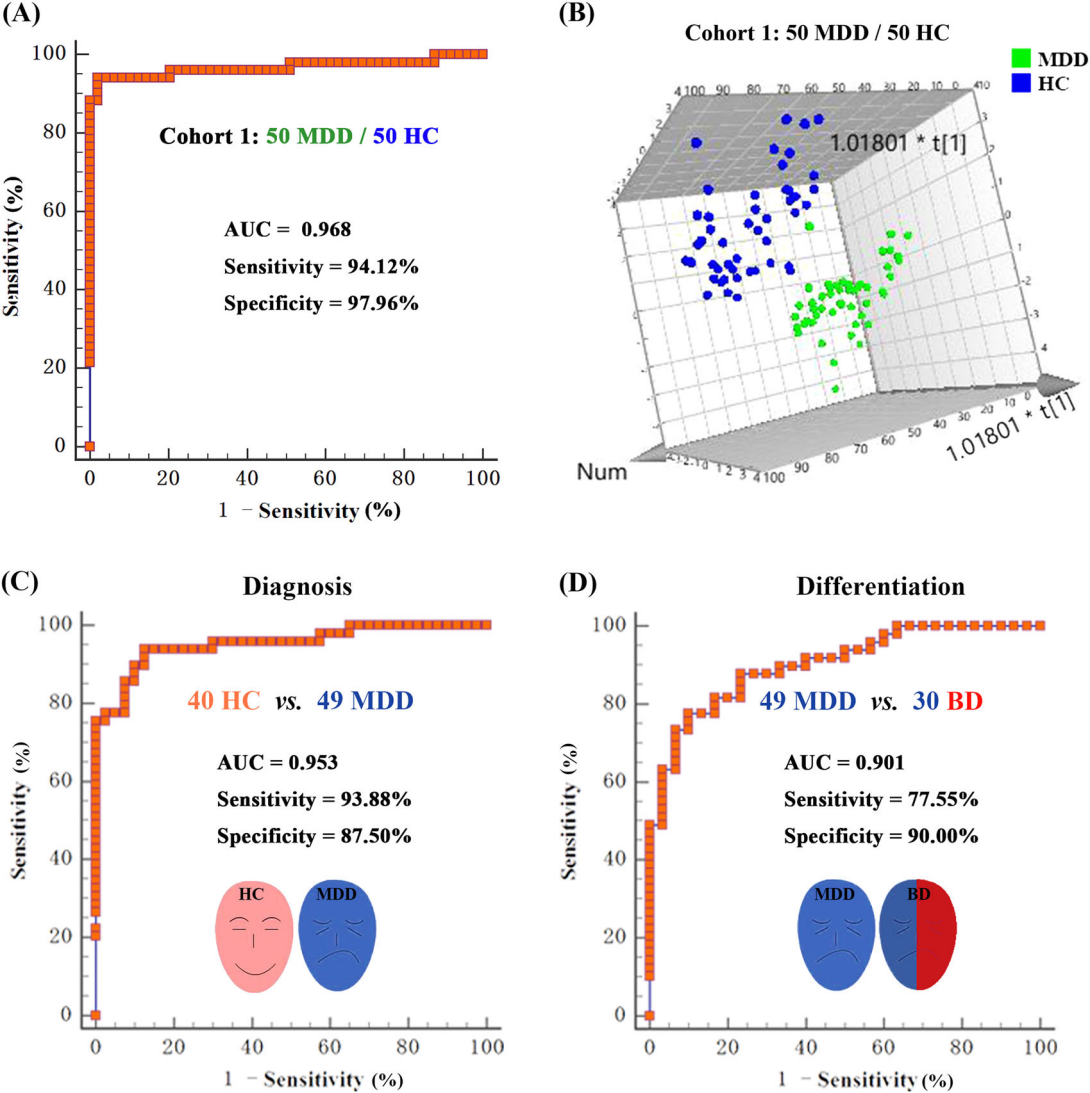
Assessment of the diagnostic perfomance of biomarker panel consisting of four metabolites (DOPN, GABA, Tyra, and Kyn). **a** ROC analysis shows that these four neurometabolite signature discriminates 50 first-episode, antidepressant drug-naïve MDD subjects and 50 healthy controls, with an area under the curve(AUC) of 0.968 in cohort 1. **b** Using the four plasma metabolites to construct the PLS-DA model, a clear discrimination between MDD subjects and HC was observed. Independent validation showing that the plasma neurometabolite biomarker panel can effectively discriminate the 49 MDD subjects from 40 healthy controls (**c**, diagnosis) and from 30 BD subjects (**d**, differential diagnosis) with an AUC of 0.953 and 0.901, respectively, in cohort 2

To obtain a simpler and more accurate biomarker panel, binary logistic regression analysis was performed to identify the optimal metabolite biomarkers. We found that a biomarker panel composed of four metabolites—DOPN, GABA, Tyra, and Kyn—could provide the most significant deviations between MDD patients and health controls, yielding an AUC of 0.968 (95% confidence interval: 0.911–0.993; Fig. 3a). To further validate the diagnostic specificity of this plasma neurometabolite signature, the identified metabolites were used to construct the PLS-DA. Consistent with the ROC analysis, a clear discrimination between 49 MDD subjects and 40 healthy controls was observed (Fig. 3b).

To independently validate the diagnostic performance of this plasma metabolite signature, 49 unselected MDD subjects, 30 BD subjects and 40 healthy controls were selected in cohort 2. The concentration of the four plasma metabolites (DOPN, GABA, Tyra, and Kyn) were independently quantified in cohort 2 (Supplemental Table 5, Supplemental Fig. 3). The ROC analysis showed that this plasma metabolite signature could effectively discriminate MDD subjects from healthy controls with an AUC of 0.953 (95% confidence interval: 0.886–0.987; Fig. 3c). To validate the diagnostic specificity of this plasma metabolite signature, ROC analysis between MDD and BD subjects was performed, which demonstrated that the 49 MDD subjects were effectively discriminated from the 30 BD subjects, with an AUC of 0.901 (95% confidence interval: 0.813–0.957; Fig. 3d).

## Discussion

MDD is a complex, heterogeneous psychiatric disorder, partly attribute to secondary effects of illness chronicity and/or antipsychotic medication. Therefore, we chose first-episode, antidepressant drug-naïve to reduce the samples heterogeneity^29^. Presently, the diagnosis of MDD remains primarily subjective. Therefore, people who are depressed are often not correctly diagnosed, and others who do not have this disorder are too often misdiagnosed and prescribed antidepressants^30,31^. A major barrier to effective care is inaccurate assessment^32^. The aim of this study was to examine the feasibility of an empirical laboratory-based method to diagnose MDD. Here, by targeted assessment of plasma metabolites from multiple neurotransmitter systems, we identified a plasma neurometabolite signature able to distinguish first-episode, antidepressant drug-naïve depressed patients from healthy controls. Moreover, this biomarker panel was able to accurately diagnose blinded samples with both high sensitivity and high specificity.

Numerous recent studies have identified hundreds of potential biomarkers for depression; however, their roles in depressive illness are unclear and they have been unable to enhance diagnosis, treatment or prognosis^33^. This lack of progress is partially due to the heterogeneity of depression, in conjunction with methodological heterogeneity within the published papers^34^. The most prominent molecular endophenotypes and biomarkers of depression are neurotransmitters, including dopamine and GABA, and components of the serotonin pathway^35–37^. This is the first report to globally evaluate multiple neurotransmitters in the plasma of MDD patients, although changes in neurotransmitter levels have been implicated in many neuropsychiatric diseases. Our previous studies also found disturbance of some neurotransmitters in MDD animal models and patients^26,38–42^. Therefore, in the present study, metabolites involved in GABA, catecholamine and tryptophan metabolism in the plasma of depressed subjects and healthy controls were assessed by targeted metabolomics to identify those that are significantly differentially expressed in MDD subjects. Furthermore, the combination of GC-MS and LC-MS/MS used here can enhance detection and overcome their individual disadvantages.

In clinical practice, BD cases are often misdiagnosed as MDD because of the similarity in clinical symptoms^43^. Recently, researchers have investigated the psychopathological characteristics of bipolar and unipolar depression^44^ and found different pathophysiologic processes underlying the depressive episodes in MDD and BD, especially in the neural circuitry regulating emotion, reward and attention^45^. Our group previously identified candidate biomarkers for diagnosing MDD^11,12,46–49^ and BD^50–53^, respectively. These biomarkers are capable of accurately distinguishing MDD and BD patients from healthy controls. However, it remained unknown whether these biomarkers can be used to differentiate MDD from BD. To address this issue, 30 BD subjects were also recruited in the current study to validate the diagnostic specificity of the biomarker panel. We found that this diagnostic panel could effectively discriminate the 49 MDD subjects from 30 BD subjects, with an AUC of 0.901 (95% confidence interval: 0.813–957).

Here, we found that plasma GABA levels in MDD subjects were increased in cohorts 1 and 2. GABA is an inhibitory transmitter that has long been associated with mental illnesses, including anxiety, depression, and schizophrenia^54–56^. Studies of patients and animal models increasingly suggest a key role for functional imbalances between the major excitatory and inhibitory neurotransmitters, including GABA and its receptors^57^. Dysregulated GABA neurotransmission in MDD has been reported in the plasma, CSF and cortex of depressed subjects^58–60^. Consistent with our results, environmental factors, including stress and excessive alcohol use, may increase GABA, causing symptoms of depression or mania^61^. Indeed, the panel of biomarkers in the GABA pathway (SA, GABA, α-KG, and Gln) effectively discriminated MDD subjects from healthy controls, with an AUC of 0.904, suggesting that perturbations in GABAergic neurotransmission may be causal for depressive disorders.

Many diseases such as depression, BD, Parkinson’s disease and attention deficit hyperactivity disorder are associated with abnormal catecholamine neurotransmitter levels. Kunugi et al.^62^ proposed a subtype of depressed patients: a dopamine-related subset of patients who present with anhedonia and respond well to aripiprazole. Indeed, the presence of such subgroups might underlie the discrepancies between previous studies, and furthermore, they highlight the need for stratified treatment. Zhao et al.^63^ reported that dopamine dysfunction in depressive patients might be a sign of diathetic depression or a depressive subtype, with medication unable to alter dopamine levels. In the present study, we systematically evaluated the plasma concentrations of dopamine and its metabolites, and found that plasma dopamine concentration was upregulated in depressed patients. This is in keeping with a previous report^64^, showing a marked increase in plasma and urinary norepinephrine in patients with major affective disorders, such as depression, bipolar depression, and unipolar depression. Furthermore, abnormal catecholaminergic neurotransmitters levels were detected in the prefrontal lobe of a depressed mouse model in our previous study^26^. Collectively, these findings suggest that plasma catecholamine neurotransmitters are comparatively reliable biological markers for MDD.

We also found altered tryptophan metabolism in MDD subjects. Numerous studies suggest that brain serotonin plays a critical role in patients with depression, and the relationship between tryptophan metabolism intermediates and depression has recently been highlighted^65,66^. Several authors have described non-targeting metabolomics methods to determine the concentration of metabolites in the 5-HT and kynurenine pathways^67,68^. Here, we used a reliable targeted metabolomic method using GC-MS combined with LC-MS/MS to quantitate Trp, Kyn, 5-HT, 5-HIAA, 3-HA, NAS, and Tra. We found that the levels of Trp and Kyn were decreased in the plasma of first-episode, antidepressant drug-naïve depressed subjects compared with healthy controls. In line with this speculation, a recent study showed that plasma metabolites related to the kynurenine pathway are downregulated during high suicidal ideation^58^. However, in this study, the diagnostic efficacy of serotonergic pathway was not good as GABAergic and catecholaminergic pathway, suggesting that single peripheral serotonergic system could not discriminate MDD from healthy controls well. Serotonergic system combined with other neurotransmitters may performed better as a biomarker in diagnosis of MDD. The biomarker panel in this study, involving three pathways, can discriminate depressed patients from healthy controls and BD subjects with high accuracy.

### Limitation

There are some limitations that should be noted in this study. First, the altered neurotransmitters identified in this study should be validated by metabolomic analysis of cerebrospinal fluid or brain tissues obtained from depressed patients. Second, as the MDD is a heterogeneous psychiatric disorder, we could not cover all the subtype. The subjects may not be very well described in clinical dimensions, although all the subjects were recruited with relatively strict criteria. Further studies using a larger sample size with more detailed clinical characteristics are required to validate the diagnostic performance. Lastly, all subjects were recruited from the same site; thus, site-specific biases cannot be ruled out. Further studies recruiting heterogeneous subjects from different clinical sites are required.

## Conclusion

In this study, using a GC-MS coupled with LC-MS/MS-based targeted metabolomics approach, we characterized changes in plasma neurotransmitter metabolites in the early stage of MDD, and identified a potential plasma diagnostic metabolite panel. This metabolite biomarker panel discriminates depressed patients from healthy controls and BD subjects with high accuracy. Our findings should contribute to uncovering the molecular pathogenesis of MDD, and they lay the foundation for the development of diagnostic and prognostic tests for the disorder.

## Electronic supplementary material

Supplementary materials
