# Supplementary material for: Diagnosis of major depressive disorder based on changes in multiple plasma neurotransmitters: a targeted metabolomics study
Source: Transl Psychiatry. 2018 Jul 10;8:130. doi: 10.1038/s41398-018-0183-x (PMC6039504; doi:10.1038/s41398-018-0183-x)
Supplement: Supplementary file 1 — Supplementary materials [file 41398_2018_183_MOESM1_ESM.doc]

**Supplementary Materials**

Supplementary materials include Extended Experimental Procedures, 5 tables and 3 figures.

Extended Experimental Procedures

**Sample preparation**

Fasting blood samples were collected into EDTA-coated tubes and centrifuged at 3000 *g* for 15 minutes at 4°C to obtain plasma. Each plasma sample was divided into equal aliquots and stored at −80 °C until use.

For GC-MS analysis, 50 uL plasma sample was added to 200 μL of methanol. After vortexing for 5 min, the mixture was subsequently centrifuged at 14,000 *g* for 10 min at 4°C. Then, 200 μL of supernatant was evaporated to dryness under a stream of nitrogen gas. The dried metabolic extract was derivatized first with 20 µL of methoxamine for 90 min at 37°C with continuous shaking. Subsequently, 40 µL of hexamethyldisiloxane (HMDS) was added to the mixture for 30 min at 40°C with continuous shaking. Thereafter, 20 µL of N-Methyl-bis-perfluorobutyramide (MBHFBA) was added and heated at 80°C for 10 min for derivatives. After derivatization and cooling to room temperature, this sample was injected into the GC/MS for analysis.

For LC-MS/MS, 50 μL of plasma was added to 215 µL of acetonitrile with 10 µL of 5µg/mL 4-aminosalicylic acid as an internal standard. After vortexing for 5 min, the mixture was centrifuged at 14,000 *g* for 10 min at 4°C. Then 220 μL supernatant was evaporated to dryness under a stream of nitrogen gas. Subsequently, 25 µL of 100 mM sodium tetraborate buffer solution and 1% benzoyl chloride solution (V/V) were added to the dried metabolic extract with continuous vortexing for 5 min. The mixture was centrifuged at 14,000 *g*, 4 °C for 10 min. A 40-µL aliquot of the supernatant was used for LC-MS/MS analysis.

**GC/MS and LC-MS acquisition**

GC/MS analysis was carried out according to our previously published work^26^. Briefly, a 1-μL volume of the derivatized sample was injected into an Agilent 7890A/5975C Inert Triple Axis Detector (Agilent, USA）. An HP-5 MS fused silica capillary column (30 m × 0.25 mm × 0.25 µm, Agilent) was used for metabolite separation with helium carrier gas at a flow rate of 1 ml/min. The injector temperature was set at 280°C. The column temperature was initially kept at 80°C for 2 min and then increased to 320°C at 10°C/min, where it was held for 6 min. The column effluent was introduced into the ion source of an Agilent 5975 mass selective detector (Agilent). The MS quadrupole temperature was set at 150°C, and the ion source temperature was set at 230°C. Data acquisition was performed first in the full-scan mode (scanning range from 50 to 550 m/z) and then in selected ion monitoring (SIM) mode for quantification. The characteristic fragment ions and retention times of metabolites were shown in supplemental Table 1.

LC-MS analysis was employed to quantify low abundance neurotransmitters, which was carried out according to our previously published work^27^. Briefly, we used the AB Sciex Triple Quad6500 mass spectrometry (MS) system with Waters ACQUITY UPLC chromatographic instrument control. The metabolites were isolated using a waters UPLC amino column (2.1 mm × 100 mm, 1.7 µm) with a maintained column temperature of 40 °C. The flow rate was set at 0.25 mL/min, and the sample volume was 5 µL. MS operating parameters were as follows: ion spray voltage, 4,500 V; source temperature, 550 °C; curtain gas, 20; CAD gas, 8; nebulizer gas (GS1), 50; auxiliary gas (GS2), 50; EP, 10; CXP, 10. The multiple reaction ronitoring (MRM) condition is shown in supplemental Table 2.

**Targeted metabolomic data analysis**

The original spectral data from GC−MS were converted to NetCDF format and then processed by XCMS software for peak finding, integration and alignment. The optimized XCMS parameters were set as follows: method = “matchedFilter”; full width at half maximum (fwhm) = 4.0; signal-to-noise cutoff (snthresh) = 10.0; retention time window (bw) = 3. Each metabolite concentration was expressed as relative abundance (metabolite peak area of study sample divided by that of the QC sample) before the following statistical analysis.

Analyst software (AB Sciex, v. 1.5.2) was used for data collection and analysis for LC-MS/MS using the default parameters for automatic identification and integration of the MRM transition. A quantitative standard curve was prepared by diluting the initial mixture of 25 analytes three times with eight gradients; this was repeated three times for LC-MS analysis. For each analysis, we chose five linear range concentrations from the eight gradients to generate the standard curve. Standard linear regression curves were drawn with the mass of the analyte-peak area on the vertical axis and with analyte concentration on the horizontal axis.

**SUPPLEMENTAL FIGURE LEGENDS**

**Figure S1. Heat map of plasma neurometabolites in cohort 2.**

Abbreviations: GABA, γ-aminobutyric acid; Tyra, tyramine; DOPN, dopamine; Kyn, kynurenine.

The heat map was generated using MetaboAnalyst 3.0 (www.metaboanalyst.ca) for each metabolite.

**Figure S2. The correlation heatmap displays the correlation coefficients (Spearman) of each molecular signature change with clinical feature in first-episode, antidepressant drug-naïve MDD patients.**

The color-coded scale of correlation is at the bottom, where a blue color indicates a positive correlation, while a red color indicates a negative correlation.

**Figure S3. AUC ROC analysis of all differential metabolites for the diagnosis of MDD subjects vs healthy controls.**

Abbreviations: SA, succinic acid; GABA, γ-aminobutyric acid; a-KG, α-ketoglutaric acid; Gln, glutamine; L -Tyr, L -tyrosine; Tyra, tyramine; DOPN, dopamine; Trp, tryptophan; Kyn, kynurenine.

**SUPPLEMENTARY TABLES**

**Table S1. The characteristic ions and retention times of metabolites**

**Table S2. MRM Conditions of the AB Sciex Triple Quad 6500 MS system**

**Table S3. AUC ROC of all differential metabolites for the diagnosis of MDD vs healthy controls**

**Table S4. AUC ROC analysis of the three pathways for the diagnosis of MDD vs healthy controls**

**Table S5. Concentration (ng/g) of plasma neurometabolites in cohort 2**

**Supplemental Figure S1**

**
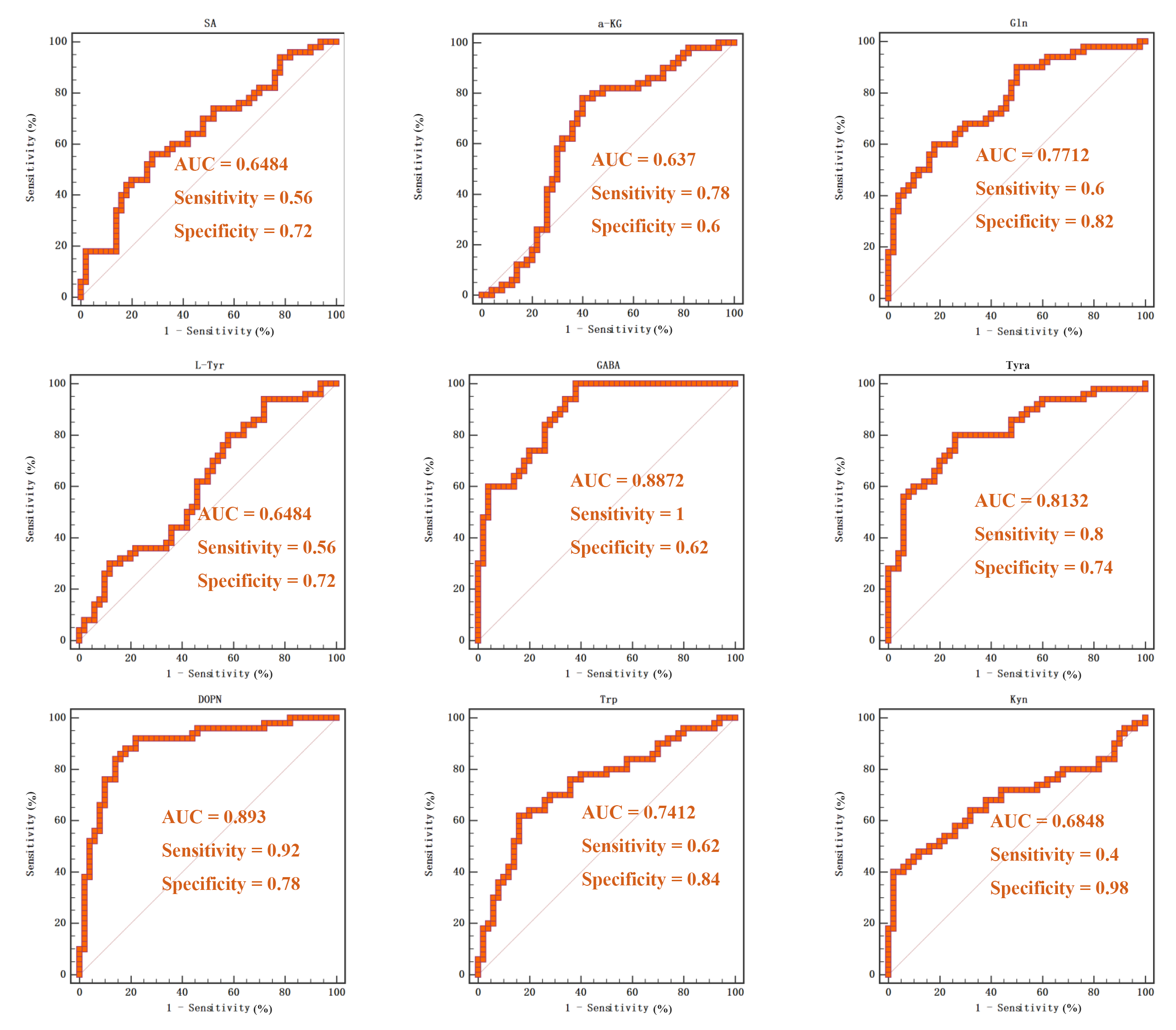
**

**Supplemental Figure S2**


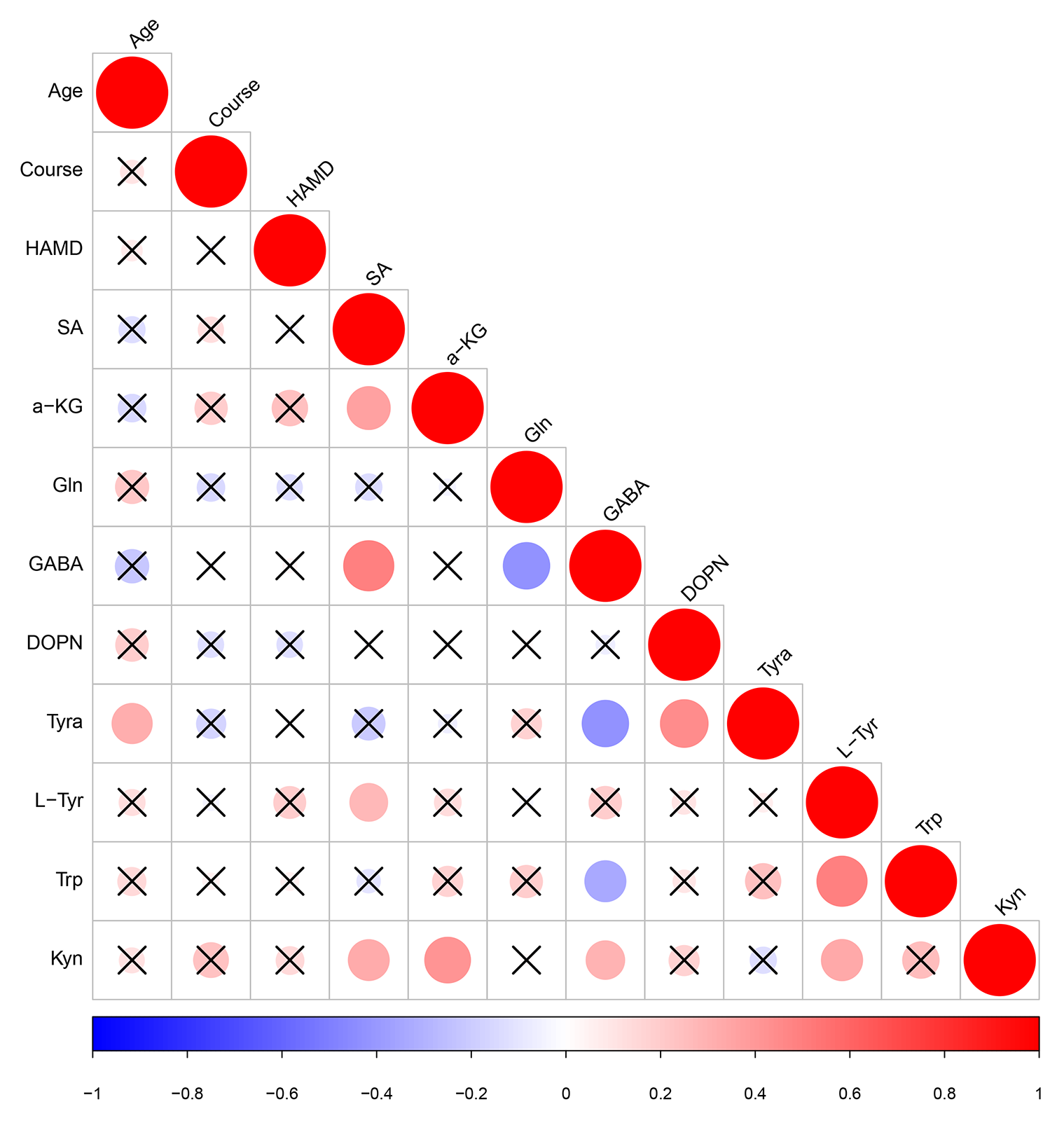


**Supplemental Figure S3**


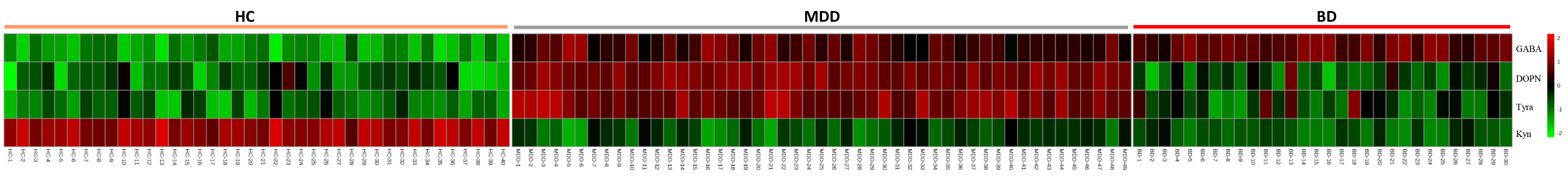


| **Supplemental Table 1. The characteristic ions and retention times of metabolites** | | | |
| --- | --- | --- | --- |
| No. | Metabolites | RT (min) | m/z |
| 1 | SA | 8.45 | 247; 172 |
| 2 | GABA | 8.95 | 356; 262 |
| 3 | a-KG | 11.77 | 198; 288 |
| 4 | Glu | 11.83 | 252; 216 |
| 5 | L-Phe | 11.94 | 91; 205 |
| 6 | Orn | 12.31 | 266; 312 |
| 7 | Gln | 13.63 | 351; 453 |
| 8 | 3-HA | 13.83 | 192; 297 |
| 9 | L-Tyr | 15.04 | 179; 308 |
| 10 | L-DOPA | 16.58 | 267; 179 |
| 11 | Trp | 17.67 | 130; 472 |
| IS | caffeic acid | 17.54 | 219; 396 |

Abbreviations: SA, succinic acid; GABA, γ-aminobutyric acid; a-KG, α-ketoglutaric acid; Glu, glutamate; L-Phe, L-phenylalanine; Orn, ornithine; Gln, glutamine; 3-HA, 3-hydroxyanthranilic acid; L -Tyr, L -tyrosine; L-DOPA, L -3,4-dihydroxyphenylalanine; Trp, tryptophan;

**Supplemental Table 2.** **MRM Conditions of the AB Sciex Triple Quad 6500 MSsystem**

| Metabolites | Retention  time (min) | Precursor  (m/z) | Product  (m/z) | Dwell  time (ms) | DP  (V) | CE  (eV) |
| --- | --- | --- | --- | --- | --- | --- |
| DOPN | 5.81 | 466.1 | 241.1 | 30 | 160 | 20 |
| 5-HIAA | 3.48 | 313.1 | 146.0 | 30 | 50 | 20 |
| Kyn | 3.43 | 417.1 | 122.1 | 30 | 60 | 21 |
| kynurenic acid | 3.56 | 294.0 | 105.1 | 30 | 50 | 20 |
| Tyra | 4.93 | 346.2 | 225.1 | 30 | 140 | 22 |
| HA | 3.71 | 304.2 | 137.1 | 30 | 50 | 18 |
| NAS | 3.44 | 323.0 | 264.3 | 30 | 120 | 24 |
| Tra | 3.54 | 265.0 | 144.0 | 30 | 80 | 18 |
| 5-HT | 4.62 | 385.0 | 264.1 | 30 | 120 | 23 |
| IS (4-aminosalicylic acid) | 3.79 | 362.2 | 256.0 | 30 | 60 | 34 |

Abbreviations: DOPN, dopamine; 5-HIAA, 5-hydroxyindoleacetic acid; Kyn, kynurenine; Tyra, tyramine; HA, Homovanillic acid; NAS, N-acetyl-serotonin; Tra, tryptamine; 5-HT, 5-hydroxytryptamine;

| **Supplemental Table 3.** **AUC ROC of all differential metabolites for the diagnosis of MDD vs healthy controls** | | | | | | |
| --- | --- | --- | --- | --- | --- | --- |
| Metabolites | Function | AUC | 95% CI | Sensitivity (%) | Specificity (%) | Youden's index |
| SA | GABAergic | 0.648 | 0.547-0.741 | 56.00 | 72.00 | 0.2800 |
| GABA | GABAergic | 0.887 | 0.808-0.942 | 100.00 | 62.00 | 0.6200 |
| α-KG | GABAergic | 0.637 | 0.538-0.734 | 78.00 | 60.00 | 0.3800 |
| Gln | GABAergic | 0.771 | 0.676-0.849 | 60.00 | 82.00 | 0.4200 |
| L-Tyr | Catecholaminergic | 0.610 | 0.507-0.706 | 80.00 | 42.00 | 0.2200 |
| Tyra | Catecholaminergic | 0.813 | 0.723-0.884 | 80.00 | 74.00 | 0.5400 |
| DOPN | Catecholaminergic | 0.893 | 0.815-0.946 | 92.00 | 78.00 | 0.7000 |
| Trp | Serotonergic | 0.741 | 0.644-0.824 | 62.00 | 84.00 | 0.4600 |
| Kyn | Serotonergic | 0.685 | 0.584-0.774 | 40.00 | 98.00 | 0.3800 |

Abbreviations: CI, confidence interval; SA, succinic acid; GABA, γ-aminobutyric acid; a-KG, α-ketoglutaric acid; Gln, glutamine; L -Tyr, L -tyrosine; Tyra, tyramine; DOPN, dopamine; Trp, tryptophan; Kyn, kynurenine.

| **Supplemental Table 4 AUC ROC analysis of the three pathways for the diagnosis of MDD vs healthy controls** | | | | | |
| --- | --- | --- | --- | --- | --- |
| Pathway | AUC | 95% CI | Sensitivity | Specificity | Youden's index |
| Catecholaminergic pathway | 0.913 | 0.839 - 0.960 | 86.27 | 89.80 | 0.7607 |
| GABAergic pathway | 0.904 | 0.829 - 0.954 | 90.20 | 75.51 | 0.6571 |
| Serotonergic pathway | 0.731 | 0.633 - 0.815 | 47.06 | 95.92 | 0.4298 |

| **Supplemental Table. 5 Concentration (ng/g) of plasma neurometabolites in cohort 2.** | | | | | | | | | | | |
| --- | --- | --- | --- | --- | --- | --- | --- | --- | --- | --- | --- |
| Metabolites | Platform | Function | HC | MDD | BD | MDD *vs.* HC | | MDD vs. BD | | BD *vs.* HC | |
|  |  |  |  |  |  | Log_2_(FC) | P value | Log_2_(FC) | P value | Log_2_(FC) | P value |
| GABA | GC-MS | GABAergic | 337.145±3.527 | 363.626±4.871 | 375.43±11.460 | 0.11 | **0.003** | -0.07 | 0.210 | 0.18 | **0.000** |
| Tyra | LC-MS/MS | Catecholaminergic | 1.455±0.168 | 5.568±0.706 | 1.989±0.418 | 2.02 | **0.000** | 1.57 | **0.000** | 0.45 | 0.522 |
| DOPN | LC-MS/MS | Catecholaminergic | 0.229±0.020 | 0.879±0.092 | 0.260±0.027 | 2.11 | **0.000** | 1.93 | **0.000** | 0.18 | 0.770 |
| Kyn | GC-MS | Serotonergic | 2153.52±71.63 | 1926.62±62.52 | 1923.51±45.61 | -0.16 | **0.000** | 0.002 | 0.830 | -0.16 | **0.000** |

Abbreviations: GABA, γ-aminobutyric acid; Tyra, tyramine; DOPN, dopamine; Kyn, kynurenine.

A negative log_2_ (FC) indicated significantly lower expression; a positive log_2_ (FC) indicated significantly higher expression.

These data were analyzed using one-way ANOVA followed by Bonferroni post hoc test between two groups of each cohort

Values given in bold denote statistically significant results (P < 0.05)
